# Transcriptome profiling reveals miR-9-3p as a novel tumor suppressor in gastric cancer

**DOI:** 10.18632/oncotarget.16310

**Published:** 2017-03-17

**Authors:** Qingshun Meng, Longquan Xiang, Jingwei Fu, Xianqun Chu, Chunlin Wang, Bingzheng Yan

**Affiliations:** ^1^ Department of Gastroenterology, Jining No.1 People's Hospital, Shandong, China; ^2^ Department of Pathology, Jining No.1 People's Hospital, Shandong, China; ^3^ Department of Gastrointestinal Surgery, Jining No.1 People's Hospital, Shandong, China; ^4^ Department of General Surgery, The First Affiliated Hospital of Xinjiang Medical University, Urumqi, Xinjiang, China

**Keywords:** gastric cancer, microarray, miR-9-3p, prognosis, cell invasion

## Abstract

It has been well established that microRNAs (miRNAs) play important roles in biological processes. To comprehensively measure the altered miRNA expression, we presented the miRNA expression profile of gastric cancer using microarray. We identified 33 miRNAs that were significantly differentially regulated in gastric specimens compared to adjacent normal tissues, among which miR-9-3p expression are significantly down-regulated in gastric cancers. Next, a cohort of 100 gastric cancer tissues and matched normal tissues were enrolled. Kaplan–Meier and multivariate Cox survival analyses were applied to evaluate the prognostic value of miR-9-3p expression, and the result showed that patients with lower miR-9-3p expression level have significantly poorer overall survival. The expression level of miR-9-3p has been proved to be an independent prognostic factor for 5-year overall survival. Furthermore, the result indicated that over-expression of miR-9-3p can inhibit gastric cancer cell invasion. Taken together, our results suggested that miR-9-3p plays important role in tumor invasion, and these findings implicated the potential effects of miR-9-3p on prognosis of gastric cancer.

## INTRODUCTION

Gastric cancer is currently one of the most frequent malignant cancers, and the second leading cause of cancer mortality in East Asian countries [1]. Most of gastric cancer patients have been diagnosed when the tumor has progressed to late tumor stages because of the nonspecific symptoms present at early stages, and the overall 5-year survival rate of gastric cancer was <20% [2]. Despite of advances in cancer treatment, limited progress has been accomplished in recent years. It is necessary to discover novel diagnostic biomarkers for the early diagnosis.

MicroRNAs (miRNAs) constitute a class of small, single-strand, non-coding RNAs containing about 22-25 nucleotides. Through the hybridization to target 3’-untranslated regions (UTR) of mRNAs, microRNA can lead to the degradation of mRNA or inhibition of translation. They are important trans-regulators of gene expression, and play pivotal role at the post-transcriptional regulation levels [3]. It has been documented that miRNAs play critical roles in many human biological processes, including cell growth, apoptosis, proliferation and differentiation [4, 5]. In recent works, studies have demonstrated that aberrantly expressed miRNAs is involved in the tumorigenesis and progression, and a number of miRNAs have been documented to regulate tumor carcinogenesis and metastasis [6, 7]. Some miRNAs have been reported to function as biomarkers for gastric cancer diagnosis and prognosis, such as miR-320a [8] and miR-371-5p [9]. These works suggested that miRNAs can act as biomarkers and represent potential therapeutic targets for treating gastric cancer [10–18].

In this work, a microarray-based genome-wide miRNA analysis was performed from 10 gastric cancer samples and matched normal tissues. We found that a total of 33 miRNAs were differentially expressed in gastric cancers (*F.D.R* < 0.05). Potential target genes and biological functions that might be affected by these miRNAs were identified by performing a bioinformatics analysis. Furthermore, we detected the underlying mechanism of miR-9-3p in gastric cancer. The expression of miR-9-3p was shown to be des-regulated in several human cancers, including breast cancer and hepatocellular carcinoma [19, 20]. We found that patients with lower miR-9-3p expression level have significantly poorer overall survival, and miR-9-3p expression was proved to be an independent prognostic factor for 5-year overall survival. Furthermore, the result indicated that over-expression of miR-9-3p can inhibit gastric cancer cell invasion. These findings suggest the potential effects of miR-9-3p on gastric cancer prognosis.

## RESULTS

### MiRNA expression is altered in gastric cancer

**Figure 1:**
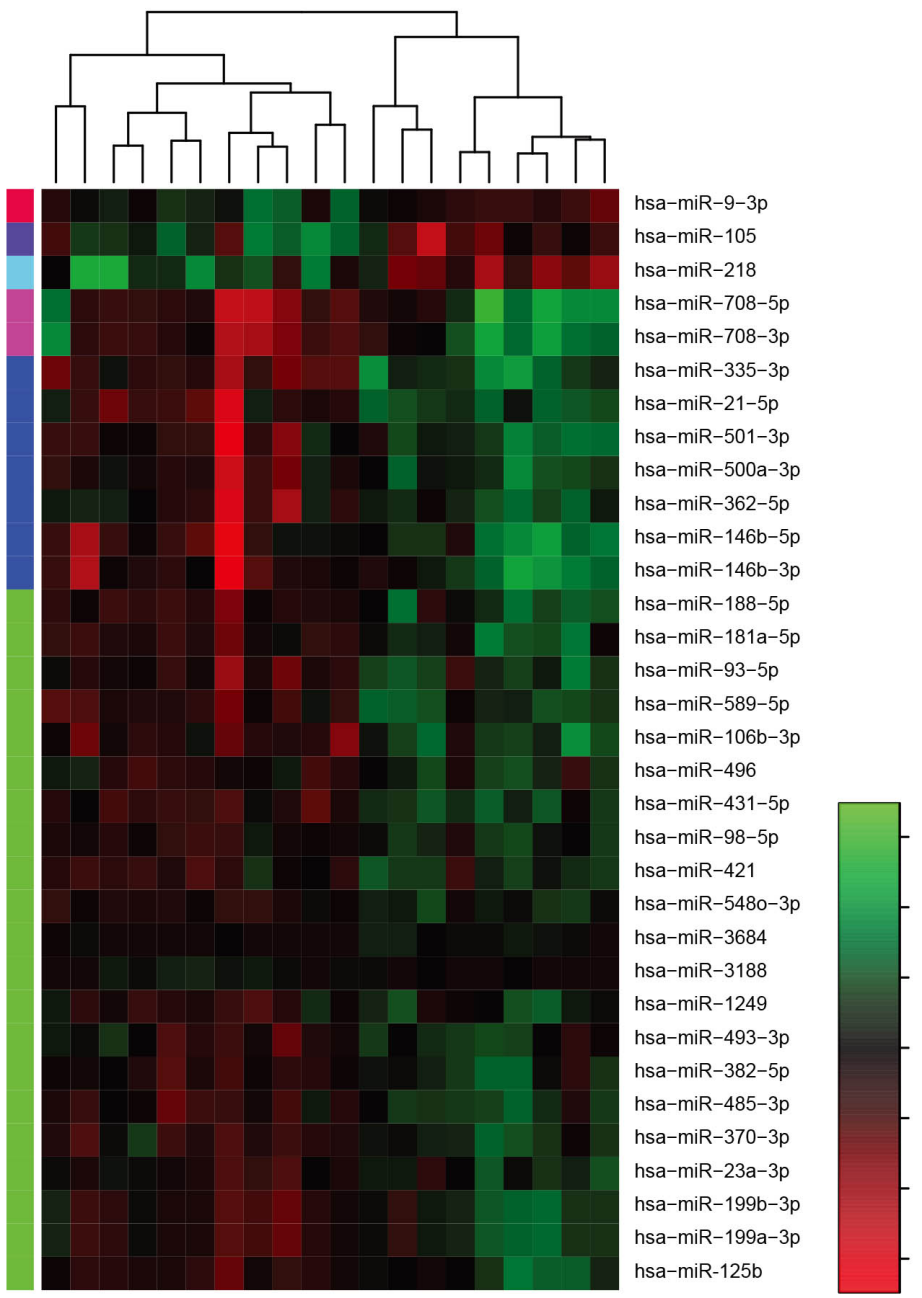
Heat map of dysregulated miRNA expression

To evaluate the miRNA expression profiles of human gastric cancer compared to those of matched normal gastric tissues, we performed a microRNA array analysis in 10 gastric tumor tissues and matched normal tissues. We detected differentially expressed miRNAs using a stringent criteria (*F.D.R*<0.01). The differentially expressed miRNAs are presented in Supplementary Table 1. We found that 33 miRNAs were differentially expressed in gastric cancer tissues, with 29 miRNAs are up-regulated and 4 miRNAs are down-regulated (Figure 1).

**Figure 2:**
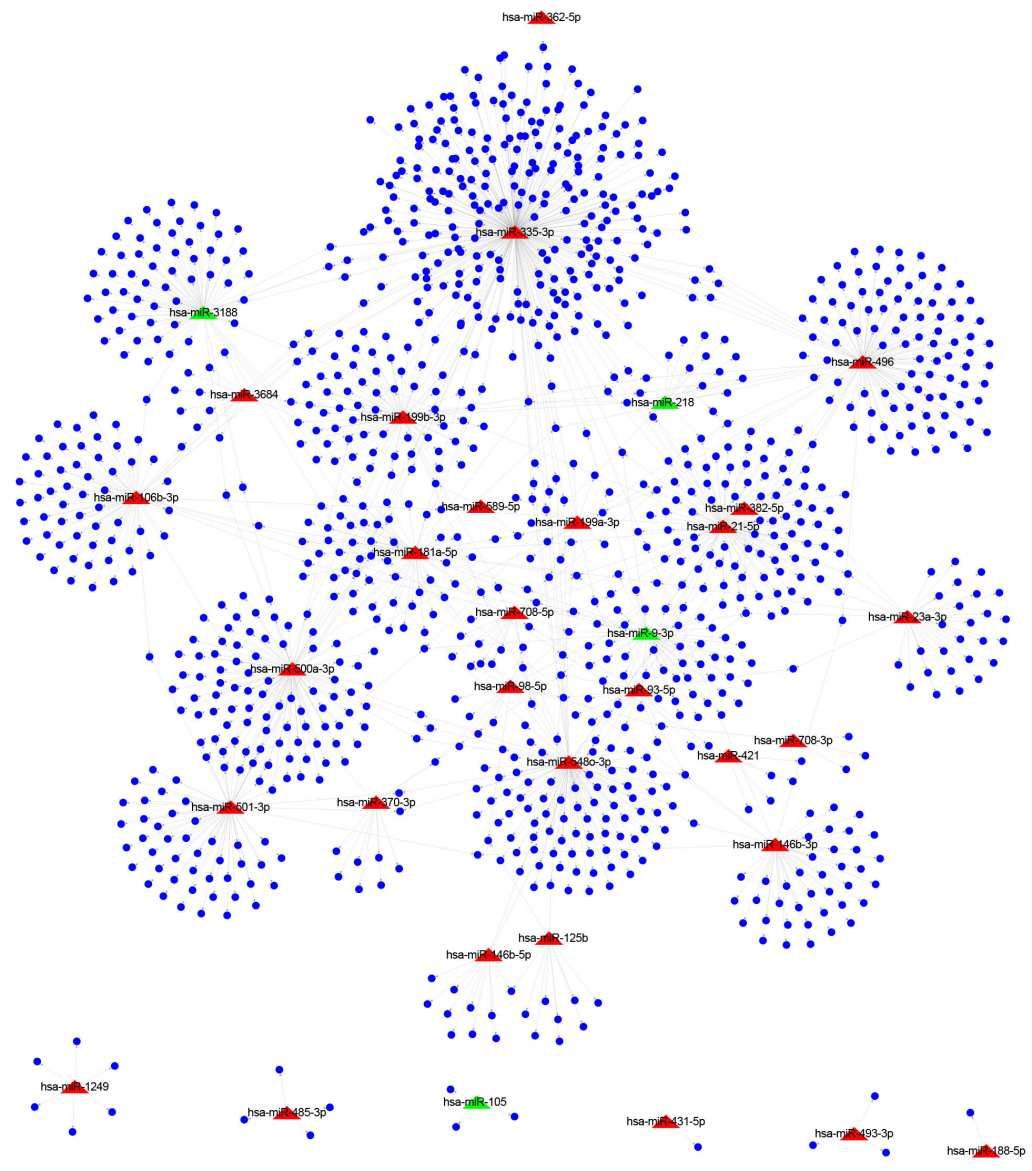
The regulatory network consisting of miRNAs and target genes Red triangles and blue nodes represent 33 differentially expressed miRNAs and 1179 target genes, respectively.

Next, target genes of dysregulated miRNAs were predicted. Here, TargetScan software [21] was employed to predict miRNA targets. TargetScan predicts potential target genes of miRNAs by searching for the presence of conserved sequences that match the seed region of each miRNA. A total of 1179 genes were identified as predicted targets of 33 differentially expressed miRNAs, and a regulatory network was constructed (Figure 2). The red and green nodes edges in the network were used to designate up- and down-regulated miRNAs in tumors, respectively. As a result, 29 up-regulated miRNAs were found to interact with 1043 target genes and 4 down-regulated miRNAs were determined to interact with 136 target genes. Among these dysregulated miRNAs, many of them have been reported to play important role in gastric cancer. For example, miR-218 was down-regulated and function as a tumor suppressor gene in gastric cancer [22]; miR-125b was up-regulated in gastric cancer tissue that might function as an oncogene in gastric cancer [23]. To examine the over-represented biological function of the predicted target genes associated with 33 differentially expressed miRNAs, we performed Gene Ontology (GO) enrichment. GO categories are organized into three different levels: biological process, molecular function and cellular component. In this study, only biological process and molecular function categories were considered. As shown in Table 1, a total of 10 GO categories are over-represented (*F.D.R*<0.05). These over-represented GO categories include “digestive system process (GO:0022600)”, “regulation of body fluid levels (GO:0050878)”, “secretion (GO:0046903)” and “digestion (GO:0007586)”.

**Table 1:** Enriched GO categories of dysregulated miRNA target genes

GO category	GO ID	GO term	*F.D.R*
BP	GO:0050878	regulation of body fluid levels	0.00022
	GO:0022600	digestive system process	0.0045
	GO:0022610	biological adhesion	0.0047
	GO:0046903	secretion	0.0058
	GO:0007155	cell adhesion	0.0068
	GO:0007586	digestion	0.0095
	GO:0045776	negative regulation of blood pressure	0.034
MF	GO:0005184	neuropeptide hormone activity	0.00083
	GO:0005509	calcium ion binding	0.00082
	GO:0003823	antigen binding	0.0026

### The expression of miR-9-3p in gastric cancer tissues and cell lines

**Figure 3:**
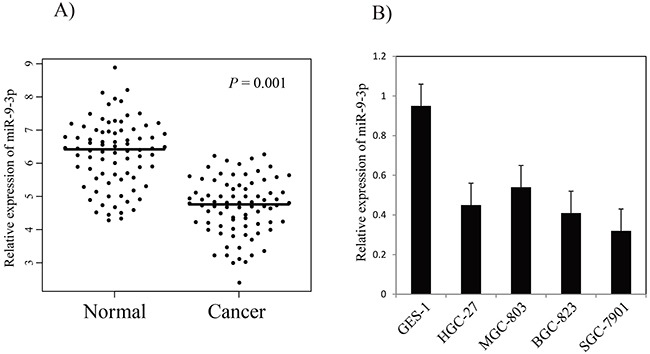
The relative expression levels of miR-9-3p in gastric cancer tissues and cell lines **(A)** the relative expression of miR-9-3p in gastric cancer tissues and adjacent non-tumor tissues. The bars represent the means of the relative expression of miR-9-3p. **(B)** the relative expression of miR-9-3p in gastric cancer cell lines.

miR-9-3p is particularly interesting because it has not been characterized in gastric cancer. We detected the expression of miR-9-3p in another cohort of 100 pairs of gastric cancer tissues and matched adjacent non-tumor tissues using qRT-PCR method. The result showed that miR-9-3p expression level was significantly lower in gastric cancer tissues (4.9 ± 1.48), compared with adjacent non-tumor tissues (6.4 ± 1.4, *P-value* = 0.001, Figure 3A). We further examined the expression of miR-9-3p expression in human gastric cancer cell lines (HGC-27, MGC-803, BGC-823 and SGC-7901), and the result indicated that miR-9-3p expressions in gastric cancer cell lines were significantly lower compared with human fetal gastric epithelial cell line (GES-1) (Figure 3B). Our work suggests that down-regulated expression of miR-9-3p is a common event in human gastric cancer tissues and might be involved in gastric cancer carcinogenesis.

### Correlation of miR-9-3p expression with clinicopathological features of gastric cancer patients

In order to evaluate the association between miR-9-3p expression and clinicopathological variables, the 100 patients with gastric cancer were divided into two groups according to the median value of miR-9-3p expression level. These two groups include high-expression group (n = 50) and low-expression group (n = 50). The correlation between miR-9-3p expression level and clinicopathological features was measured. As shown in Table 2, the result showed that lower expression of miR-9-3p was significantly associated with higher incidence of lymph node metastasis (*P-value* = 0.004). However, no significant correlations between miR-9-3p expression and other characteristics of patients were observed.

**Table 2:** Clinicopathological associations of miR-9-3p expression in gastric cancer patients

miR-9-3p expression			
Variable	High (n=50)	Low (n=50)	*P-value*
Ages (years)			0.81
< 50	24	22	
≥ 50	26	28	
Gender			
Male	34	30	0.66
Female	16	20	
Tumor size			0.78
< 2 cm	24	22	
≥ 2 cm	26	28	
Tumor location			
Upper third	10	13	0.47
Middle third	8	7	
Lower third	12	10	
Histological grading			0.14
Poorly differentiated	33	27	
Well differentiated	17	23	
Lymph node metastasis			0.004
Absent	34	22	
Present	16	28	
Clinical stage			0.078
I + II	32	24	
III	18	26	

**Figure 4:**
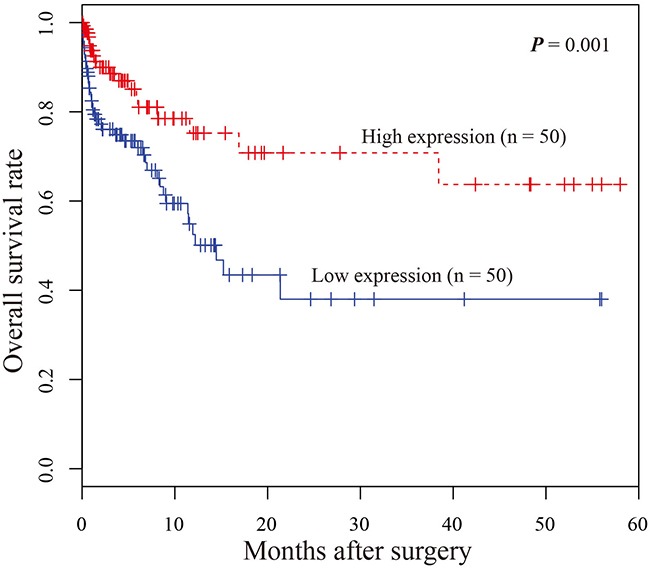
Kaplan–Meier survival curves of patients with gastric cancer based on miR-9-3p expression status Patients with low expression group have significantly poorer prognosis than those in high expression group.

Next, Kaplan–Meier survival analysis was performed to examine the relationship between miR-9-3p expression with survival of gastric cancer patients. The results showed that gastric cancer patients with lower miR-9-3p expression level had a significantly poorer prognosis than those with high miR-9-3p expression level (*P-value* = 0.001). It suggested that down-regulation of miR-9-3p might be associated with poor survival of gastric cancer patients (Figure 4). Univariate proportional hazard model indicated that miR-9-3p expression level was prognostic predictors of gastric cancer patients. The result was further evaluated using multivariate analysis, and the result demonstrated that miR-9-3p was independent prognostic factor for overall survival (*P-value* = 0.001, Table 3).

**Table 3:** Univariate and multivariate analyses of prognostic factors in gastric cancer patients

Variables	Univariate analysis			Multivariate analysis		
	HR	95% CI	*P-value*	HR	95% CI	*P-value*
Age	1.53	0.73-2.01	0.64	1.28	0.76-1.81	0.44
Gender	1.33	0.87-1.87	0.38	1.22	0.67-1.34	0.34
Lymph node metastasis	1.18	0.78-1.75	0.21	1.18	0.78-1.54	0.15
Tumor size	2.28	1.66-2.84	0.36	1.56	0.90-2.18	0.51
Clinical stage	1.73	1.15-2.66	0.082	1.42	1.22-2.08	0.12
miR-9-3p	2.66	1.86-3.85	<0.001	1.56	1.11-2.86	0.001

### *In vitro* effect of miR-9-3p on gastric cancer cell invasion

**Figure 5:**
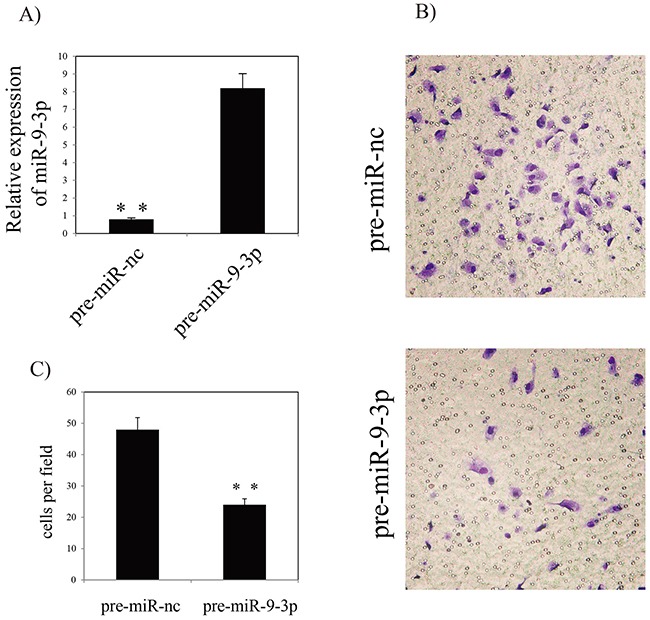
Effects of miR-9-3p on invasion of HGC-27 cell line **(A)** RT-PCR analysis of miR-9-3p in HGC-27 cells **(B)** Invasion assay of HGC-27 cells and representative fields of invasive cells. **(C)** Average number of invasive cells per field from three independent experiments. ** indicates P<0.01

**Figure 6:**
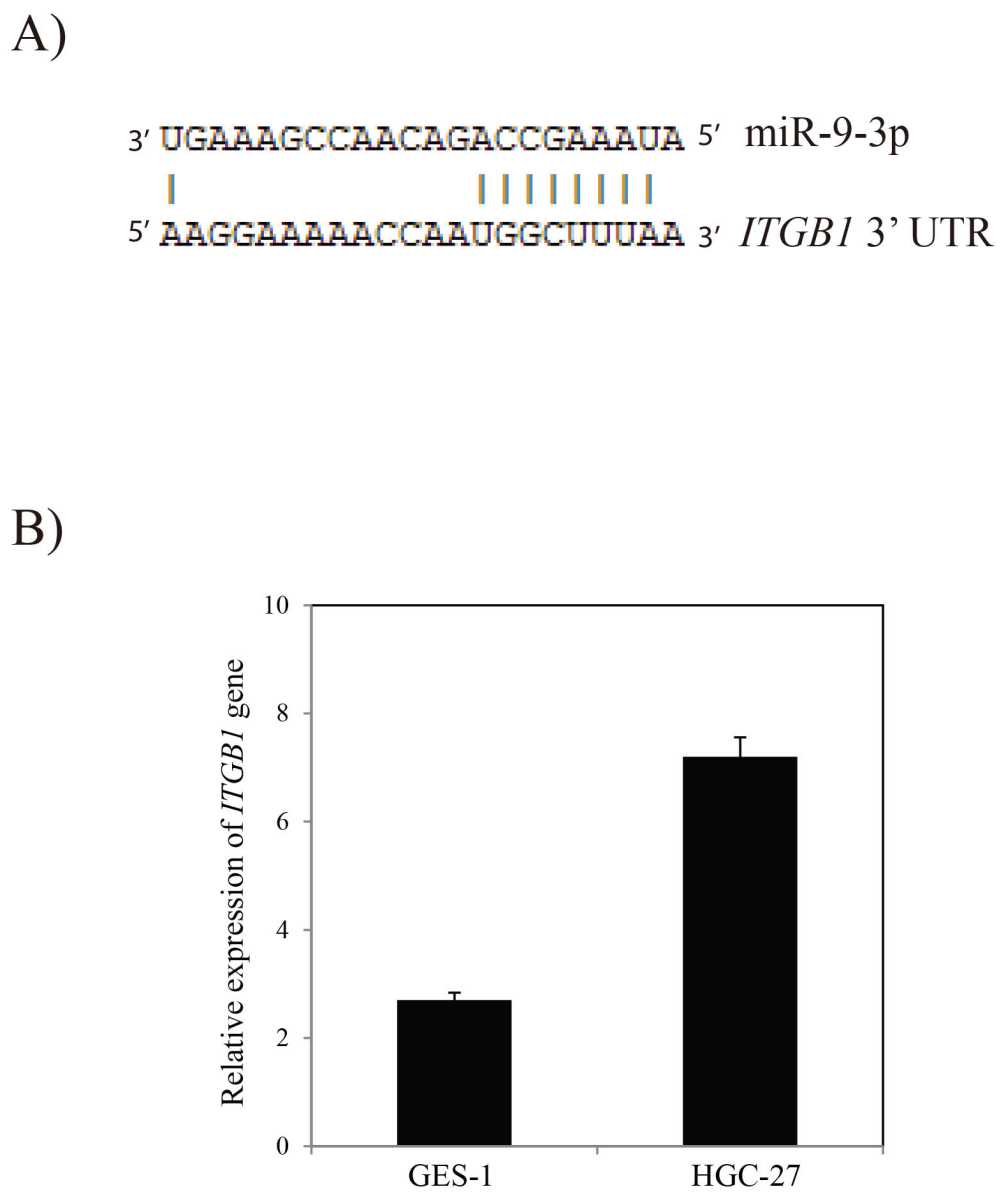
The *ITGB1* gene is the target of miR-9-3p **(A)** Alignment of miR-9-3p with 3’ UTR of *ITGB1* gene. **(B)** RT-PCR analysis of *ITGB1* gene in HGC-27 cell line.

To assess the functional effects of miR-9-3p *in vitro*, we investigated whether miR-9-3p regulates gastric cancer cell invasion using a Transwell assay. *In vitro* gastric cancer cell invasion assays was performed by transfecting pre- miR-9-3p or pre-miR negative control (pre-miR-nc) into human HGC-27 cell. The result showed that over-expression of miR-9-3p significantly inhibits gastric cancer cell invasion (Figure 5). Our result showed that miR-9-3p can bind to the 3’ UTR of the *ITGB1* transcript. The result showed that seven base pairs of identity were observed at both putative target sites (Figure 6A). In order to confirm the potential relationship between miR-9-3p and *ITGB1* gene, we detected the expression level of *ITGB1* in different cell lines using RT-PCR. The result demonstrated that the expression of *ITGB1* is increased in HGC-27 cell line (Figure 6B).

## DISCUSSION

Gastric cancer is one of the leading cause of cancer-related death worldwide. Identifying the biomarker of gastric cancer is crucial to select optimal therapeutic strategies [12, 24]. Recent studies have shown the dysregulation of miRNAs in gastric cancer and focused on detecting specific biological markers with prognosis in gastric cancer [25]. As a novel biomarker, miRNAs have been implicated in many steps of tumor development and progression [26–30]. Detecting cancer-specific miRNAs and their binding targets is important to understand their roles in tumorigenesis [31–33]. In the present work, we performed a miRNA microarray work to identify dysregulated miRNAs in gastric cancer tissues compared to those found in adjacent normal tissues. Our study provided many dysregulated miRNAs and some of them may have clinical use and act as diagnostic and prognostic biomarkers.

Some previous works also performed microRNA expression profiling in gastric cancer [34, 35], and these studies provided many differentially expressed miRNAs. Then, we measured the consistently and inconsistently reported dysregulated microRNA. Most of the miRNAs were reported to be with consistent direction of expression compared with previous results, except miR-9-3p. Previous work has been reported that down-regulation of miR-9-3p plays important functions in medullary thyroid carcinoma [36], and miR-9-3p might function as a tumor suppressor gene. However, Previous transcriptome data shows no differential expression of miR-9-3p in gastric cancer. To further validate our result, we examined the miR-9-3p expression in gastric cancer tissues and adjacent non-tumor tissues in a different cohort. Furthermore, we examined the association of miR-9-3p expression and clinicopathologic features and prognosis of gastric cancer patients. The results showed that the relative expression of miR-9-3p was significantly lower in gastric cancer tissues and cell lines. Interestingly, multivariate survival analysis showed that low expression of miR-9-3p is involved in gastric cancer and might be used as an independent potential prognostic biomarker for gastric cancer patients. Further analysis showed that down-regulation of miR-9-3p could significantly inhibit cell invasion, which indicates that miR-9-3p might function as a tumor suppressor in gastric cancer.

Taken together, the current work indicated that the expression of miR-9-3p was significantly down-regulated in gastric cancers and is tightly associated with cancer cell invasion. These results implicated for the first time that miR-9-3p expression might be an important modulator involved in gastric cancer.

## MATERIALS AND METHODS

### Patient samples collection

Surgical specimens of gastric cancer tissues and matched non-cancerous normal tissues were obtained from 110 patients with a diagnosis of gastric cancer who underwent surgery at the first people's Hospital of Jining (Shandong Province, China) between June 2007 and April 2014. The patients recruited in this study had never received radiotherapy before surgery excision. This work was approved by the ethics committee of the first people's Hospital of Jining, and informed consents were obtained from each patient. All tissue samples were immediately frozen in liquid nitrogen after surgical removal and stored at -80°C until RNA extraction.

### miRNA microarray and miRNA target prediction

The total RNA was extracted from tissues or cell lines using the TRIzol Reagent (Invitrogen, Carlsbad, California, USA) following the manufacturer's protocol. A total of 20 tissue specimen (10 gastric cancer samples and matched normal tissues) were used for a microRNA array analysis. The analysis (miRNA3.0 Array; DNALink) was performed using 2 μg of total RNA. TargetScan (version 6.2) [21] was used to identify targets genes of differentially expressed miRNAs. The comprehensive regulatory network of miRNAs and their target genes was generated used Cytoscape software.

### quantitative RT-PCR

We performedquantitative RT-PCR using a miR-9-3p qRT-PCR Detection Kit (Stratagene Corp, La Jolla, CA). The expression of U6 was selected as an internal control. Relative quantification of miR-9-3p expression was measured by using the 2^-ΔΔCT^ method.

### Cell culture and transfection of miRNA

Four human gastric cancer cell lines (HGC-27, MGC-803, BGC-823 and SGC-7901) and human fetal gastric epithelial cell line (GES-1) were purchased from the Type Culture Collection cell bank of the Chinese Academy of Sciences (Shanghai, China) and cultured in Dulbecco's modified Eagle's medium (Gibco, USA) supplemented with 10% fetal bovine serum. Cells were incubated at 37 °C in a humidified atmosphere containing 5% CO_2_. The pre-miR miR-9-3p (Pre-miR-9-3p), pre-miR negative control (Pre-miR-nc) were purchased from Applied Biosystems (ABI, Foster City, CA, USA). Transfection of miR-9-3p was performed using Lipofectamine^TM^ 2000 Reagent (Invitrogen) following the manufacturer's instructions.

### *In vitro* invasion assays

Cell invasion assay was performed using transwell chambers (BD, Biosciences). Matrigel was coated on the upper compartment of the transwell chamber before use. Medium containing 10% fetal bovine serum in the lower chamber was used as the chemoattractant. After 48 hours incubation, the non-invaded cells were removed from the top chambers. The number of cells on the lower surface of the membrane were quantified under a microscope field in five random fields.

### Statistical analysis

In this work, the differences between different groups were assessed using Mann-Whitney U test or Chi-square test. Kaplan–Meier curves were analyzed to assess the overall survival rate. A Cox proportional hazards regression model was used to evaluate the association between the potential prognostic marker and overall survival. All statistical analyses were performed using R package. The differences were considered to be statistically significant at a threshold P-value of < 0.05 (two sided P-values).

### Ethical statements

Patients were identified from a prospective institutional database and the study was approved by the local Research Ethics Committee.

## SUPPLEMENTARY MATERIALS AND TABLES





## References

[1] Siegel RL, Miller KD, Jemal A (2015). Cancer statistics, 2015. CA Cancer J Clin.

[2] Tang H, Kong Y, Guo J, Tang Y, Xie X, Yang L, Su Q, Xie X (2013). Diallyl disulfide suppresses proliferation and induces apoptosis in human gastric cancer through Wnt-1 signaling pathway by up-regulation of miR-200b and miR-22. Cancer Lett.

[3] Bartel DP (2004). MicroRNAs: genomics, biogenesis, mechanism, and function. Cell.

[4] Croce CM, Calin GA (2005). miRNAs, cancer, and stem cell division. Cell.

[5] Gregory RI, Shiekhattar R (2005). MicroRNA biogenesis and cancer. Cancer Res.

[6] Lu J, Getz G, Miska EA, Alvarez-Saavedra E, Lamb J, Peck D, Sweet-Cordero A, Ebert BL, Mak RH, Ferrando AA, Downing JR, Jacks T, Horvitz HR, Golub TR (2005). MicroRNA expression profiles classify human cancers. Nature.

[7] Volinia S, Calin GA, Liu CG, Ambs S, Cimmino A, Petrocca F, Visone R, Iorio M, Roldo C, Ferracin M, Prueitt RL, Yanaihara N, Lanza G (2006). A microRNA expression signature of human solid tumors defines cancer gene targets. Proc Natl Acad Sci USA.

[8] Wang Y, Zeng J, Pan J, Geng X, Li L, Wu J, Song P, Liu J, Wang L (2016). MiR-320a inhibits gastric carcinoma by targeting activity in the FoxM1-P27KIP1 axis. Oncotarget.

[9] Li YJ, Dong M, Kong FM, Zhou JP, Liang D, Xue HZ (2016). MicroRNA-371-5p targets SOX2 in gastric cancer. Oncotarget.

[10] Wu ZS, Wang CQ, Xiang R, Liu X, Ye S, Yang XQ, Zhang GH, Xu XC, Zhu T, Wu Q (2012). Loss of miR-133a expression associated with poor survival of breast cancer and restoration of miR-133a expression inhibited breast cancer cell growth and invasion. BMC Cancer.

[11] Zheng TH, Zhao JL, Guleng B (2015). Advances in Molecular Biomarkers for Gastric Cancer. Crit Rev Eukaryot Gene Expr.

[12] Irmak-Yazicioglu MB (2016). Mechanisms of MicroRNA Deregulation and MicroRNA Targets in Gastric Cancer. Oncol Res Treat.

[13] Ibarrola-Villava M, Llorca-Cardenosa MJ, Tarazona N, Mongort C, Fleitas T, Perez-Fidalgo JA, Rosello S, Navarro S, Ribas G, Cervantes A (2015). Deregulation of ARID1A, CDH1, cMET and PIK3CA and target-related microRNA expression in gastric cancer. Oncotarget.

[14] Li Z, Yu X, Wang Y, Shen J, Wu WK, Liang J, Feng F (2015). By downregulating TIAM1 expression, microRNA-329 suppresses gastric cancer invasion and growth. Oncotarget.

[15] Tsai MM, Huang HW, Wang CS, Lee KF, Tsai CY, Lu PH, Chi HC, Lin YH, Kuo LM, Lin KH (2016). MicroRNA-26b inhibits tumor metastasis by targeting the KPNA2/c-jun pathway in human gastric cancer. Oncotarget.

[16] Chang S, He S, Qiu G, Lu J, Wang J, Liu J, Fan L, Zhao W, Che X (2016). MicroRNA-125b promotes invasion and metastasis of gastric cancer by targeting STARD13 and NEU1. Tumour biology.

[17] Yin K, Liu M, Zhang M, Wang F, Fen M, Liu Z, Yuan Y, Gao S, Yang L, Zhang W, Zhang J, Guo B, Xu J (2016). miR-208a-3p suppresses cell apoptosis by targeting PDCD4 in gastric cancer. Oncotarget.

[18] Li C, Song L, Zhang Z, Bai XX, Cui MF, Ma LJ (2016). MicroRNA-21 promotes TGF-beta1-induced epithelial-mesenchymal transition in gastric cancer through up-regulating PTEN expression. Oncotarget.

[19] Higashi T, Hayashi H, Ishimoto T, Takeyama H, Kaida T, Arima K, Taki K, Sakamoto K, Kuroki H, Okabe H, Nitta H, Hashimoto D, Chikamoto A (2015). miR-9-3p plays a tumour-suppressor role by targeting TAZ (WWTR1) in hepatocellular carcinoma cells. Br J Cancer.

[20] Zawistowski JS, Nakamura K, Parker JS, Granger DA, Golitz BT, Johnson GL (2013). MicroRNA 9-3p targets beta1 integrin to sensitize claudin-low breast cancer cells to MEK inhibition. Mol Cell Biol.

[21] Lewis BP, Burge CB, Bartel DP (2005). Conserved seed pairing, often flanked by adenosines, indicates that thousands of human genes are microRNA targets. Cell.

[22] Ruan Q, Fang ZY, Cui SZ, Zhang XL, Wu YB, Tang HS, Tu YN, Ding Y (2015). Thermo-chemotherapy Induced miR-218 upregulation inhibits the invasion of gastric cancer via targeting Gli2 and E-cadherin. Tumour biology.

[23] Li X, Zhang Y, Zhang H, Liu X, Gong T, Li M, Sun L, Ji G, Shi Y, Han Z, Han S, Nie Y, Chen X (2011). miRNA-223 promotes gastric cancer invasion and metastasis by targeting tumor suppressor EPB41L3. Molecular cancer research.

[24] Liu D, Hu X, Zhou H, Shi G, Wu J (2014). Identification of Aberrantly Expressed miRNAs in Gastric Cancer. Gastroenterology research and practice.

[25] Yang TS, Yang XH, Wang XD, Wang YL, Zhou B, Song ZS (2013). MiR-214 regulate gastric cancer cell proliferation, migration and invasion by targeting PTEN. Cancer Cell Int.

[26] Chen P, Zhao X, Ma L (2013). Downregulation of microRNA-100 correlates with tumor progression and poor prognosis in hepatocellular carcinoma. Mol Cell Biochem.

[27] Giordano S, Columbano A (2013). MicroRNAs: new tools for diagnosis, prognosis, and therapy in hepatocellular carcinoma?. Hepatology.

[28] Gu H, Guo X, Zou L, Zhu H, Zhang J (2013). Upregulation of microRNA-372 associates with tumor progression and prognosis in hepatocellular carcinoma. Mol Cell Biochem.

[29] Huang XY, Yao JG, Huang HD, Wang C, Ma Y, Xia Q, Long XD (2013). MicroRNA-429 Modulates Hepatocellular Carcinoma Prognosis and Tumorigenesis. Gastroenterology research and practice.

[30] Kitagawa N, Ojima H, Shirakihara T, Shimizu H, Kokubu A, Urushidate T, Totoki Y, Kosuge T, Miyagawa S, Shibata T (2013). Downregulation of the microRNA biogenesis components and its association with poor prognosis in hepatocellular carcinoma. Cancer Sci.

[31] Kim M, Kasinski AL, Slack FJ (2011). MicroRNA therapeutics in preclinical cancer models. Lancet Oncol.

[32] Patnaik SK, Kannisto E, Knudsen S, Yendamuri S (2010). Evaluation of microRNA expression profiles that may predict recurrence of localized stage I non-small cell lung cancer after surgical resection. Cancer Res.

[33] Liu A, Tetzlaff MT, Vanbelle P, Elder D, Feldman M, Tobias JW, Sepulveda AR, Xu X (2009). MicroRNA expression profiling outperforms mRNA expression profiling in formalin-fixed paraffin-embedded tissues. Int J Clin Exp Pathol.

[34] Kim CH, Kim HK, Rettig RL, Kim J, Lee ET, Aprelikova O, Choi IJ, Munroe DJ, Green JE (2011). miRNA signature associated with outcome of gastric cancer patients following chemotherapy. BMC Med Genomics.

[35] Li X, Luo F, Li Q, Xu M, Feng D, Zhang G, Wu W (2011). Identification of new aberrantly expressed miRNAs in intestinal-type gastric cancer and its clinical significance. Oncol Rep.

[36] Chen Y, Zhang S, Zhao R, Zhao Q, Zhang T (2016). Upregulated miR-9-3p promotes cell growth and inhibits apoptosis in medullary thyroid carcinoma by targeting BLCAP. Oncol Res.

