# Supplementary material for: Transcriptome profiling reveals miR-9-3p as a novel tumor suppressor in gastric cancer
Source: Oncotarget. 2017 Mar 17;8(23):37321–31. doi: 10.18632/oncotarget.16310 (PMC5514911; doi:10.18632/oncotarget.16310)
Supplement: Supplementary file 1 [file oncotarget-08-37321-s001.pdf]

Transcriptome profiling reveals miR-9-3p as a novel tumor suppressor in gastric cancer

SUPPLEMENTARY TABLE

Supplementary Table 1: The expression level of dysregulated miRNAs.

See Supplementary File 1
