# Supplementary material for: Transcriptome profiling reveals miR-9-3p as a novel tumor suppressor in gastric cancer
Source: Oncotarget. 2017 Mar 17;8(23):37321–31. doi: 10.18632/oncotarget.16310 (PMC5514911; doi:10.18632/oncotarget.16310)
Supplement: Supplementary file 2 [file oncotarget-08-37321-s002.docx]

Table S1 The expression level of dysregulated miRNAs

| **miRNA_name** | ***F.D.R*** | **tumor_mean_expression** | **normal_mean_expression** |  |
| --- | --- | --- | --- | --- |
| hsa-miR-21-5p | 3.65E-06 | 232616.1235 | 75525.64437 | up-regulated |
| hsa-miR-431-5p | 0.00041279 | 2.5389242 | 0.5627717 | up-regulated |
| hsa-miR-589-5p | 0.001023162 | 69.0967478 | 29.6544985 | up-regulated |
| hsa-miR-199b-3p | 0.001195576 | 2372.731886 | 1025.703645 | up-regulated |
| hsa-miR-199a-3p | 0.001196134 | 2376.933166 | 1027.590568 | up-regulated |
| hsa-miR-485-3p | 0.001348889 | 5.1016481 | 2.0837623 | up-regulated |
| hsa-miR-93-5p | 0.001426938 | 3161.935559 | 1331.21459 | up-regulated |
| hsa-miR-708-3p | 0.001747928 | 56.09133 | 10.8260772 | up-regulated |
| hsa-miR-362-5p | 0.002022054 | 15.3542144 | 7.1611159 | up-regulated |
| hsa-miR-3684 | 0.002357865 | 0.2322487 | 0.0318786 | up-regulated |
| hsa-miR-98-5p | 0.003197868 | 55.2379358 | 33.8576075 | up-regulated |
| hsa-miR-335-3p | 0.003432272 | 64.3249386 | 16.540589 | up-regulated |
| hsa-miR-501-3p | 0.003462671 | 58.706049 | 22.6141187 | up-regulated |
| hsa-miR-421 | 0.004119479 | 2.7268901 | 1.0820103 | up-regulated |
| hsa-miR-106b-3p | 0.004496636 | 296.5678097 | 111.8086579 | up-regulated |
| hsa-miR-188-5p | 0.004708044 | 6.062064 | 2.0186218 | up-regulated |
| hsa-miR-146b-5p | 0.005190583 | 730.0937099 | 227.1792087 | up-regulated |
| hsa-miR-370-3p | 0.005198272 | 5.0060792 | 2.3148403 | up-regulated |
| hsa-miR-708-5p | 0.005706086 | 44.0353333 | 7.0511469 | up-regulated |
| hsa-miR-1249 | 0.005894795 | 1.897881 | 0.6459623 | up-regulated |
| hsa-miR-125b | 0.006243533 | 538.2920613 | 249.6402196 | up-regulated |
| hsa-miR-548o-3p | 0.006724865 | 0.9065956 | 0.2810969 | up-regulated |
| hsa-miR-500a-3p | 0.006895724 | 300.480826 | 131.6720824 | up-regulated |
| hsa-miR-23a-3p | 0.007034141 | 3005.292649 | 1859.828112 | up-regulated |
| hsa-miR-493-3p | 0.007803982 | 5.2437213 | 2.8879858 | up-regulated |
| hsa-miR-496 | 0.008144266 | 1.6297651 | 0.6147703 | up-regulated |
| hsa-miR-146b-3p | 0.008723518 | 105.7558622 | 31.3579624 | up-regulated |
| hsa-miR-382-5p | 0.009065843 | 19.1931216 | 9.901817 | up-regulated |
| hsa-miR-181a-5p | 0.00969875 | 796.2757902 | 350.5638192 | up-regulated |
| hsa-miR-105 | 0.001209622 | 3284.685019 | 10064.50333 | down-regulated |
| **hsa-miR-9-3p** | 0.002979802 | 0.7696034 | 2.9005136 | down-regulated |
| hsa-miR-3188 | 0.004294237 | 0.0535009 | 0.2888478 | down-regulated |
| hsa-miR-218 | 0.007160638 | 3.7412971 | 20.2823822 | down-regulated |

| **miRNA_name** | **Tumor1** | **Normal1** |
| --- | --- | --- |
| hsa-miR-21-5p | 141032.42 | 45099.204 |
| hsa-miR-431-5p | 1.82021 | 0.200469 |
| hsa-miR-589-5p | 53.117062 | 35.282618 |
| hsa-miR-199b-3p | 3657.1345 | 1009.9649 |
| hsa-miR-199a-3p | 3665.5737 | 1011.1677 |
| hsa-miR-485-3p | 9.101055 | 3.207509 |
| hsa-miR-93-5p | 3505.5606 | 1694.3675 |
| hsa-miR-708-3p | 56.757483 | 11.426755 |
| hsa-miR-362-5p | 24.821058 | 12.429102 |
| hsa-miR-3684 | 0.330948 | 0 |
| hsa-miR-98-5p | 79.261909 | 29.869944 |
| hsa-miR-335-3p | 125.76002 | 32.476045 |
| hsa-miR-501-3p | 108.55075 | 30.070412 |
| hsa-miR-421 | 3.805896 | 0.801878 |
| hsa-miR-106b-3p | 291.89563 | 146.94408 |
| hsa-miR-188-5p | 10.921265 | 1.403285 |
| hsa-miR-146b-5p | 1217.0592 | 202.27364 |
| hsa-miR-370-3p | 7.446317 | 3.40798 |
| hsa-miR-708-5p | 40.210113 | 8.219246 |
| hsa-miR-1249 | 3.309474 | 0.801877 |
| hsa-miR-125b | 1069.4566 | 255.79898 |
| hsa-miR-548o-3p | 0.661895 | 0.200469 |
| hsa-miR-500a-3p | 644.52011 | 197.06144 |
| hsa-miR-23a-3p | 2489.7175 | 2508.0729 |
| hsa-miR-493-3p | 7.446317 | 6.214552 |
| hsa-miR-496 | 2.978527 | 0.400939 |
| hsa-miR-146b-3p | 237.9512 | 44.504212 |
| hsa-miR-382-5p | 34.584007 | 13.43145 |
| hsa-miR-181a-5p | 974.80563 | 371.6703 |
| hsa-miR-105 | 4658.085 | 7807.8829 |
| **hsa-miR-9-3p** | 0.496421 | 2.205163 |
| hsa-miR-3188 | 0.165474 | 0.400939 |
| hsa-miR-218 | 4.302317 | 44.103271 |

| **miRNA_name** | **Tumor2** | **Normal2** |
| --- | --- | --- |
| hsa-miR-21-5p | 272303.86 | 70173.455 |
| hsa-miR-431-5p | 1.13221 | 0.331496 |
| hsa-miR-589-5p | 79.820749 | 31.657895 |
| hsa-miR-199b-3p | 2372.167 | 1244.2713 |
| hsa-miR-199a-3p | 2376.8845 | 1249.9068 |
| hsa-miR-485-3p | 2.075718 | 0.662992 |
| hsa-miR-93-5p | 5899.1873 | 1147.1429 |
| hsa-miR-708-3p | 61.705403 | 14.917332 |
| hsa-miR-362-5p | 23.587692 | 6.464178 |
| hsa-miR-3684 | 0.188702 | 0 |
| hsa-miR-98-5p | 42.269145 | 26.188206 |
| hsa-miR-335-3p | 47.93019 | 18.232296 |
| hsa-miR-501-3p | 86.80271 | 19.558281 |
| hsa-miR-421 | 2.830524 | 0.994488 |
| hsa-miR-106b-3p | 659.51186 | 118.34418 |
| hsa-miR-188-5p | 9.057674 | 1.325985 |
| hsa-miR-146b-5p | 834.24948 | 194.25683 |
| hsa-miR-370-3p | 3.019225 | 1.325984 |
| hsa-miR-708-5p | 56.610462 | 9.613392 |
| hsa-miR-1249 | 1.509613 | 0.497244 |
| hsa-miR-125b | 782.16786 | 278.78838 |
| hsa-miR-548o-3p | 1.509613 | 0.165748 |
| hsa-miR-500a-3p | 333.24691 | 125.47135 |
| hsa-miR-23a-3p | 3002.0527 | 2044.6691 |
| hsa-miR-493-3p | 2.264418 | 1.325985 |
| hsa-miR-496 | 0.188702 | 0.331496 |
| hsa-miR-146b-3p | 147.7533 | 30.994901 |
| hsa-miR-382-5p | 8.30287 | 5.635436 |
| hsa-miR-181a-5p | 1454.7001 | 274.64468 |
| hsa-miR-105 | 4595.8258 | 12133.759 |
| **hsa-miR-9-3p** | 0.377404 | 2.983465 |
| hsa-miR-3188 | 0 | 0.497244 |
| hsa-miR-218 | 2.453122 | 11.105125 |

| **miRNA_name** | **Tumor3** | **Normal3** |
| --- | --- | --- |
| hsa-miR-21-5p | 185028.6 | 71703.501 |
| hsa-miR-431-5p | 2.30459 | 0.534958 |
| hsa-miR-589-5p | 39.178026 | 32.632379 |
| hsa-miR-199b-3p | 2023.2652 | 1589.6248 |
| hsa-miR-199a-3p | 2026.722 | 1590.6947 |
| hsa-miR-485-3p | 5.761476 | 2.942264 |
| hsa-miR-93-5p | 1097.3139 | 1734.0632 |
| hsa-miR-708-3p | 45.762569 | 12.036535 |
| hsa-miR-362-5p | 12.839859 | 8.826792 |
| hsa-miR-3684 | 0.164614 | 0 |
| hsa-miR-98-5p | 32.922713 | 38.516908 |
| hsa-miR-335-3p | 46.585636 | 19.525932 |
| hsa-miR-501-3p | 41.318001 | 28.352724 |
| hsa-miR-421 | 0.493841 | 0.534958 |
| hsa-miR-106b-3p | 127.74012 | 115.01577 |
| hsa-miR-188-5p | 3.456885 | 1.872351 |
| hsa-miR-146b-5p | 262.72323 | 303.58812 |
| hsa-miR-370-3p | 4.773793 | 6.152008 |
| hsa-miR-708-5p | 22.552057 | 9.09427 |
| hsa-miR-1249 | 0.987682 | 0.267479 |
| hsa-miR-125b | 350.13303 | 281.3874 |
| hsa-miR-548o-3p | 0.329227 | 0.267479 |
| hsa-miR-500a-3p | 209.38844 | 159.14972 |
| hsa-miR-23a-3p | 2253.3949 | 2416.1335 |
| hsa-miR-493-3p | 6.584542 | 3.7447 |
| hsa-miR-496 | 0.658455 | 0 |
| hsa-miR-146b-3p | 77.203756 | 35.307166 |
| hsa-miR-382-5p | 23.210512 | 10.164186 |
| hsa-miR-181a-5p | 427.99524 | 413.52179 |
| hsa-miR-105 | 2672.0072 | 5902.181 |
| **hsa-miR-9-3p** | 0.658455 | 1.337395 |
| hsa-miR-3188 | 0 | 0.267479 |
| hsa-miR-218 | 5.103021 | 11.234098 |

| **miRNA_name** | **Tumor4** | **Nomal4** |
| --- | --- | --- |
| hsa-miR-21-5p | 303848.31 | 97782.307 |
| hsa-miR-431-5p | 4.241058 | 0 |
| hsa-miR-589-5p | 60.267656 | 47.665263 |
| hsa-miR-199b-3p | 3268.5158 | 1034.4024 |
| hsa-miR-199a-3p | 3277.4444 | 1034.7334 |
| hsa-miR-485-3p | 6.473194 | 3.972106 |
| hsa-miR-93-5p | 2701.3302 | 1602.4135 |
| hsa-miR-708-3p | 107.58893 | 11.585306 |
| hsa-miR-362-5p | 19.196365 | 12.247324 |
| hsa-miR-3684 | 0.223214 | 0 |
| hsa-miR-98-5p | 72.321188 | 39.059036 |
| hsa-miR-335-3p | 104.46394 | 12.909342 |
| hsa-miR-501-3p | 54.240891 | 46.01022 |
| hsa-miR-421 | 2.45535 | 1.655045 |
| hsa-miR-106b-3p | 272.54373 | 124.4593 |
| hsa-miR-188-5p | 5.357126 | 2.317062 |
| hsa-miR-146b-5p | 909.37196 | 386.61824 |
| hsa-miR-370-3p | 9.37497 | 1.986053 |
| hsa-miR-708-5p | 111.16034 | 8.937236 |
| hsa-miR-1249 | 1.116069 | 1.986053 |
| hsa-miR-125b | 554.90886 | 250.57364 |
| hsa-miR-548o-3p | 0.892855 | 0.662018 |
| hsa-miR-500a-3p | 256.69557 | 221.44487 |
| hsa-miR-23a-3p | 4916.7246 | 2313.7513 |
| hsa-miR-493-3p | 8.035689 | 2.64807 |
| hsa-miR-496 | 2.45535 | 0 |
| hsa-miR-146b-3p | 117.18711 | 39.721053 |
| hsa-miR-382-5p | 33.035605 | 8.937238 |
| hsa-miR-181a-5p | 1555.3519 | 313.1343 |
| hsa-miR-105 | 1868.0741 | 18014.821 |
| **hsa-miR-9-3p** | 0.446428 | 0.993027 |
| hsa-miR-3188 | 0 | 0 |
| hsa-miR-218 | 0.892855 | 24.494649 |

| **miRNA_name** | **Tumor5** | **Normal5** | **Tumor6** | **Normal6** |
| --- | --- | --- | --- | --- |
| hsa-miR-21-5p | 156010.73 | 53610.994 | 228119.64 | 71203.977 |
| hsa-miR-431-5p | 0.576092 | 0.432889 | 2.85971 | 1.17884 |
| hsa-miR-589-5p | 18.764129 | 28.570674 | 67.203186 | 19.768227 |
| hsa-miR-199b-3p | 1990.3144 | 1191.7434 | 2373.5593 | 841.14715 |
| hsa-miR-199a-3p | 1994.8409 | 1194.6293 | 2375.8471 | 843.41415 |
| hsa-miR-485-3p | 2.386666 | 1.298667 | 6.005391 | 1.45088 |
| hsa-miR-93-5p | 1988.7507 | 1662.0052 | 3000.4078 | 1368.7231 |
| hsa-miR-708-3p | 78.924559 | 20.345783 | 53.476578 | 14.327431 |
| hsa-miR-362-5p | 11.274936 | 5.916149 | 11.43884 | 13.239271 |
| hsa-miR-3684 | 0.082299 | 0 | 0 | 0.18136 |
| hsa-miR-98-5p | 33.660212 | 23.231709 | 70.348867 | 36.090617 |
| hsa-miR-335-3p | 10.287352 | 20.201486 | 40.893853 | 16.41307 |
| hsa-miR-501-3p | 18.023439 | 19.480005 | 81.787707 | 18.04531 |
| hsa-miR-421 | 0.905287 | 0.288593 | 3.431652 | 0.4534 |
| hsa-miR-106b-3p | 154.80406 | 188.01812 | 328.00874 | 61.934402 |
| hsa-miR-188-5p | 1.810573 | 2.020149 | 7.721217 | 3.083118 |
| hsa-miR-146b-5p | 193.4022 | 139.53456 | 971.72948 | 74.992313 |
| hsa-miR-370-3p | 2.222068 | 2.308741 | 5.71942 | 1.08816 |
| hsa-miR-708-5p | 43.947566 | 11.255114 | 54.620461 | 9.884114 |
| hsa-miR-1249 | 1.563678 | 0.721482 | 1.143884 | 0.4534 |
| hsa-miR-125b | 333.06328 | 238.08895 | 481.2892 | 234.31697 |
| hsa-miR-548o-3p | 0 | 0.432888 | 0.571942 | 0.4534 |
| hsa-miR-500a-3p | 114.55994 | 134.48418 | 279.67964 | 144.36247 |
| hsa-miR-23a-3p | 2468.059 | 1920.7285 | 4453.4265 | 1818.2235 |
| hsa-miR-493-3p | 4.773331 | 2.308741 | 3.431652 | 3.989918 |
| hsa-miR-496 | 1.399081 | 0.432889 | 2.001797 | 1.632239 |
| hsa-miR-146b-3p | 27.652401 | 47.473494 | 117.24811 | 8.614596 |
| hsa-miR-382-5p | 12.262521 | 10.389336 | 21.447825 | 11.244314 |
| hsa-miR-181a-5p | 312.73548 | 383.39536 | 465.5608 | 277.66199 |
| hsa-miR-105 | 2652.4906 | 7028.3858 | 2791.9349 | 8341.1945 |
| **hsa-miR-9-3p** | 0.822988 | 2.453036 | 0 | 1.72292 |
| hsa-miR-3188 | 0 | 0.577185 | 0 | 0 |
| hsa-miR-218 | 7.406894 | 27.849193 | 1.143884 | 46.518811 |

| **miRNA_name** | **Tumor7** | **Normal7** | **Tumor8** | **Normal8** | **Tumor9** | **Normal9** | **Tumor10** | **Normal10** |
| --- | --- | --- | --- | --- | --- | --- | --- | --- |
| hsa-miR-21-5p | 293212.28 | 90609.214 | 293459.67 | 157609.2 | 251995.06 | 15954.621 | 201150.67 | 81509.973 |
| hsa-miR-431-5p | 3.704946 | 0.797208 | 2.378666 | 1.323342 | 2.42172 | 0.137426 | 3.95004 | 0.691089 |
| hsa-miR-589-5p | 84.073776 | 26.507151 | 104.42346 | 38.376929 | 96.428533 | 14.429742 | 87.690897 | 21.654107 |
| hsa-miR-199b-3p | 3778.19 | 1307.0218 | 1537.0944 | 762.68633 | 916.51138 | 670.08968 | 1810.5669 | 606.08462 |
| hsa-miR-199a-3p | 3781.04 | 1308.2176 | 1538.7594 | 763.56856 | 918.49279 | 671.87622 | 1813.7269 | 607.69716 |
| hsa-miR-485-3p | 5.129926 | 1.993019 | 3.092267 | 2.646685 | 5.724068 | 1.511687 | 5.26672 | 1.151814 |
| hsa-miR-93-5p | 2372.0205 | 1312.2036 | 3499.4943 | 1571.2485 | 4098.8731 | 507.92688 | 3456.417 | 712.05153 |
| hsa-miR-708-3p | 101.45852 | 9.765792 | 13.082666 | 3.970027 | 20.694706 | 3.435652 | 21.461885 | 6.450159 |
| hsa-miR-362-5p | 16.529759 | 5.979057 | 10.941866 | 4.852256 | 14.090013 | 0.274852 | 8.821756 | 1.382178 |
| hsa-miR-3684 | 0.284996 | 0 | 0.475734 | 0 | 0.440312 | 0.137426 | 0.131668 | 0 |
| hsa-miR-98-5p | 47.879304 | 35.675039 | 45.908265 | 49.404783 | 68.688816 | 23.912142 | 59.118939 | 36.627691 |
| hsa-miR-335-3p | 72.103951 | 12.55602 | 115.84106 | 17.644564 | 36.986286 | 5.771896 | 42.3971 | 9.675239 |
| hsa-miR-501-3p | 74.098924 | 31.091095 | 24.262399 | 21.614593 | 59.001934 | 2.47367 | 38.973732 | 9.444877 |
| hsa-miR-421 | 1.994971 | 0.797208 | 4.757334 | 3.087799 | 3.302346 | 0.824556 | 3.2917 | 1.382178 |
| hsa-miR-106b-3p | 212.8919 | 50.821982 | 192.90986 | 118.6597 | 289.94607 | 69.262754 | 435.42612 | 124.6263 |
| hsa-miR-188-5p | 6.554904 | 2.590925 | 3.330134 | 3.087799 | 10.56751 | 0.412278 | 1.843352 | 2.073266 |
| hsa-miR-146b-5p | 1515.038 | 347.78179 | 325.87732 | 212.1759 | 535.86085 | 28.859482 | 535.62548 | 381.71122 |
| hsa-miR-370-3p | 5.699918 | 2.19232 | 3.805867 | 2.205571 | 3.522502 | 1.099409 | 4.476712 | 1.382177 |
| hsa-miR-708-5p | 76.663884 | 6.178359 | 11.4176 | 1.764456 | 12.769074 | 1.649113 | 10.401773 | 3.916169 |
| hsa-miR-1249 | 3.704947 | 0.99651 | 1.902935 | 0 | 3.082188 | 0.274852 | 0.65834 | 0.460726 |
| hsa-miR-125b | 780.88863 | 303.93538 | 377.97012 | 299.95761 | 344.54488 | 137.01382 | 308.49816 | 216.54107 |
| hsa-miR-548o-3p | 1.42498 | 0.398604 | 0.951467 | 0 | 0.880625 | 0 | 1.843352 | 0.230363 |
| hsa-miR-500a-3p | 352.53987 | 146.48689 | 181.96799 | 100.13291 | 430.62606 | 25.698679 | 201.58373 | 62.428329 |
| hsa-miR-23a-3p | 3470.3945 | 1866.6615 | 2703.3546 | 1416.4175 | 2750.4148 | 1145.0343 | 1545.3875 | 1148.5891 |
| hsa-miR-493-3p | 3.704946 | 2.989528 | 6.660267 | 3.087799 | 3.742659 | 1.649113 | 5.793392 | 0.921452 |
| hsa-miR-496 | 1.709975 | 0.398604 | 0.951467 | 0.882228 | 1.320937 | 0.68713 | 2.63336 | 1.382178 |
| hsa-miR-146b-3p | 192.6572 | 44.643625 | 35.917866 | 21.614592 | 45.132077 | 3.847931 | 58.855602 | 36.858054 |
| hsa-miR-382-5p | 13.679802 | 12.157415 | 14.509866 | 12.351195 | 13.649699 | 6.184174 | 17.248509 | 8.523426 |
| hsa-miR-181a-5p | 947.04122 | 333.03345 | 506.41812 | 437.1441 | 700.75804 | 179.89077 | 617.39132 | 521.54146 |
| hsa-miR-105 | 3075.1052 | 12001.362 | 7429.7651 | 15544.862 | 2291.8288 | 2251.3144 | 811.7333 | 11619.271 |
| **hsa-miR-9-3p** | 0.854988 | 5.181851 | 1.665068 | 5.734483 | 1.320938 | 1.786538 | 1.053344 | 4.607258 |
| hsa-miR-3188 | 0 | 0.199302 | 0.237867 | 0.441114 | 0 | 0.274852 | 0.131668 | 0.230363 |
| hsa-miR-218 | 1.42498 | 5.580453 | 10.466134 | 15.880109 | 1.981408 | 11.681219 | 2.238356 | 4.376894 |
